# Metabolism of NK cells during viral infections

**DOI:** 10.3389/fimmu.2023.1064101

**Published:** 2023-01-18

**Authors:** Kenia Y. Osuna-Espinoza, Adrián G. Rosas-Taraco

**Affiliations:** Faculty of Medicine, Department of Immunology, Universidad Autonoma de Nuevo Leon, Monterrey, Nuevo Leon, Mexico

**Keywords:** natural killer cells (NK cells), viral infection, glycolysis, oxidative phosphorylation, immunometabolism

## Abstract

Cellular metabolism is essential for the correct function of immune system cells, including Natural Killer cells (NK). These cells depend on energy to carry out their effector functions, especially in the early stages of viral infection. NK cells participate in the innate immune response against viruses and tumors. Their main functions are cytotoxicity and cytokine production. Metabolic changes can impact intracellular signals, molecule production, secretion, and cell activation which is essential as the first line of immune defense. Metabolic variations in different immune cells in response to a tumor or pathogen infection have been described; however, little is known about NK cell metabolism in the context of viral infection. This review summarizes the activation-specific metabolic changes in NK cells, the immunometabolism of NK cells during early, late, and chronic antiviral responses, and the metabolic alterations in NK cells in SARS-CoV2 infection. The modulation points of these metabolic routes are also discussed to explore potential new immunotherapies against viral infections.

## Introduction

Natural Killer (NK) cells are innate lymphoid cells (ILCs) that share certain morphological characteristics with adaptive immunity lymphocytes, establishing a complementary innate counterpart to T helper cells. The differences between these populations are based on stimulation and the response to stimuli. The ILCs are divided into two functional groups: cytotoxic ILCs (conventional NK cells) and helper-like ILCs (ILC1, ILC2, and ILC3) (1).

NK cells are derived from a common lymphoid progenitor (CLP) and acquire their maturation state and functional capacity through the expression of diverse surface receptors, which contribute to identifying and classifying these cells (2, 3). These cells are identified by the expression of CD56/CD16 and comprise 5–15% of human peripheral blood mononuclear cells (PBMCs) (4, 5).

The NK cell classification currently accepted by the scientific community is based on the expression of the adhesion molecule CD56, establishing two functionally different phenotypes: CD56^bright^ NK cells, which participate mainly as cytokine-producing cells, and CD56^dim^ NK cells which have greater cytotoxicity (3). Other proposed classifications are based on location (tissue and circulating NK cells), expression of inhibitory receptors (educated and uneducated NK cells), or energy metabolism (basal or active states) (6, 7).

NK cells are considered the first line of immune defense because they can destroy target cells without priming. The primary functions attributed to NK cells are antitumor and antiviral activity mediated by diverse cytotoxic pathways: the release of cytotoxic granules (granzymes and perforins), induction of apoptosis by binding to death ligands, cytokine release such as interferon-γ (IFN-γ) and tumor necrosis factor-α (TNF-α), which are involved in innate and adaptive immune responses (8).

NK cell response modulation depends on integrating signals from NK cell receptors (NKRs) expressed on the cell surface and in response to different cytokines (IL-15, IL-18, IL-2). NKRs can be classified into activating and inhibitory receptors. Activating NKRs (NKG2D, NKp30, CD226, etc.) recognize specific activating ligands to transduce activating signals. On the other hand, inhibitory NKRs (NKG2A, KIR2DL, etc.) mainly detect major histocompatibility complex class I (MHC-I) molecules on potential target cells and transduce inhibitory signals to antagonize activating signals. NKR are encoded in the germ line and show great variability in their expression; therefore, two NK cells from the same individual show differences in the expression of their receptors and respond to different stimuli (8).

Once NK cells are activated, they generate well-characterized metabolic changes to satisfy the new energy requirement of the cell. This review discusses what is currently known about the different activation-specific metabolic changes in the NK cell response to viral infections and the possible control points of these metabolic routes as potential immunotherapies.

## Activation-specific metabolic changes in NK cells

NK cells are ready to kill target cells and produce cytokines upon activation. This fast response requires various metabolic changes known as “metabolic reprogramming” for optimal cell activation. There are two primary and overlapping metabolic pathways for generating adenosine triphosphate (ATP): anaerobic glycolysis and mitochondrial oxidative phosphorylation (OXPHOS). Glycolysis converts glucose into pyruvate *via* different metabolic reactions. This is oxygen-dependent pathway, and it is relatively inefficient generating just two molecules of ATP as the final product. On the other hand, OXPHOS, is an oxygen-dependent pathway that produces an estimated of 30 ATP molecules. Therefore, metabolism can be defined as the anabolic and catabolic reactions performed to obtain the energy required for cell functions (9).

All cells can sense the availability of nutrients in their microenvironment to activate different metabolic pathways according to their energy requirements. This “sensing” action is made possible by different metabolic regulators that can switch on or off metabolic pathways and thus change cellular metabolism (10).

Some “master regulators of metabolism” have been identified. One example is an AMP-protein kinase (AMPK). This protein is activated under conditions of energy deprivation where ATP levels decrease and AMP increases. Once activated, AMPK decreases energy consumption (anabolism) and increases energy production (catabolism) through the phosphorylation of key proteins in multiple pathways (11, 12).

Extensive evidence has demonstrated the role of this regulatory protein in the metabolic reprogramming of immune system cells (13–17). In NK cells of old donors, its activation negatively regulates the functional capacity of these cells, mainly in mature and terminally differentiated NK cells. These findings suggest that inhibitory receptor signaling may orchestrate AMPK-dependent metabolic pathways to decrease NK cell function, evidenced by a relationship between senescence and energy-sensing pathways (18).

Cellular metabolism is now identified as a key component of immune system cell function. Metabolic modifications can determine the phenotype of innate immune cells, such as macrophages, which has been evaluated in several *in vitro* and *in vivo* models in mice (14, 19–23). Proposing interesting hypotheses about the metabolic reprogramming of these cells to change their phenotype in different inflammatory diseases (24–26).

Investigations in the metabolism of NK cells have been developed, showing metabolic changes according to their functional phenotype, activation stimulus and activation state. In functional phenotype, NK cells with cytotoxic activity (CD56^dim^) maintain higher rates of glycolysis and OXPHOS than cytokine-producing NK cells (CD56^bright^). Also, NK cells from different tissues are functionally different according to their location and show different metabolic profiles (7, 27).

Another factor is the activation stimulus (NKRs or cytokines). *In vitro* studies, with 6-hours stimulation, have shown that IFN-γ production through receptor activation (NK1.1 or Ly49D) depends almost completely on glycolysis and OXPHOS. While in cytokine-stimulated (IL-12/IL-18) murine NK cells, IFN-γ is produced even with the blockade of multiple metabolic fuels (glucose, glutamine, fatty acids). This finding suggest an important link between activation stimulus and metabolic profile for cell effector function (28).

IL-15 is widely used to prime NK cells due to induced cell proliferation and has been reported to activate the mTOR signaling pathway, directly affecting cell metabolism (29). A recent report demonstrated that IL-15 is necessary to maintain NK cell function of receptor-activated cells, even when oxidative metabolism is blocked (28).

Finally, the activation state (resting cells, short-term and long-term activation). Murine NK cells in a resting state show relatively low rates of glycolysis and OXPHOS. However, these low metabolic rates are sufficient to sustain acute NK cell responses in terms of cytokine production. Interestingly, after short-term activation (4–6 h) with cytokines (IL-12, IL-15) or through NKR (NK1.1 and Ly49D), these cells can exert their effector functions without inducing metabolic rate changes (28).

On the other hand, in human blood NK cells, longer periods of activation enhance effector functions associated with metabolic changes. Both glycolysis and OXPHOS upregulation, supported by increased expression of glycolytic enzymes and nutrient transporters (7, 28, 30–33). These metabolic changes have also been described in other contexts of NK cell activation, including cancer, viral infections, and bacterial infections such as sepsis, where NK cell play an important role (34).

The high expression of nutrient transporters is related with increased energy requirements after cell activation. The well-studied transporters are CD98 (amino acid transporter), GLUT1 (glucose transporter), and CD71 (transferrin transporter), which transport the main nutrients required as energy substrates (7). CD98 is an amino acid transporter encoded by *Slc3a2* and forms disulfide-linked heterodimers. One of the light chains is L-type amino acid transporter 1 (LAT1), preferentially imports large neutral amino acids such as leucine, isoleucine, and valine (33). GLUT1 is one of the glucose transporters into cells and expressed on all human cells. The expression of these nutrient transporters differs between NK cell subsets in peripheral blood, which is related to the functional capacity of these cells. CD71 is a cell surface receptor required for receptor-mediated endocytosis of iron. Recently, the upregulation of these receptors has been reported on cytokine-stimulated NK cells (7).

Understanding that upregulation in metabolic pathways (glycolysis and OXPHOS) is an important characteristic of activated NK cells, several studies have evaluated the functional effect of inhibiting metabolic pathways (8, 28, 35). Most studies have used inhibitory drugs such as oligomycin (OXPHOS inhibitor), rapamycin (mTOR inhibitor), and 2-deoxyglucose (2-DG, competitive inhibition of glucose) to assess this effect (36).

It has been demonstrated that glucose metabolism inhibition directly affects the cytotoxic function of human NK cells. Glycolysis inhibition by 2-DG decreases the expression of the degranulation marker CD107a and granzyme B secretion; also, inhibition of OXPHOS by oligomycin decreases IFN-γ production (8, 28).

The mammalian target of rapamycin (mTOR) is a serine/threonine kinase which constitutes the catalytic subunit of two distinct complexes: mTOR complex 1 (mTORC1) and mTOR complex 2 (mTORC2). These complexes are distinguished by their accessory proteins and their differential functions; mTORC1 controls cell growth, proliferation, autophagy, and metabolism, while mTORC2 controls cell migration, apoptosis, and metabolism (37, 38).

In NK cells, mTOR has an important role in regulating cellular metabolism and maintaining a high rate of glycolysis to sustain their effector functions (30). In this context, various studies have focused on evaluating the effect of mTOR inhibition on NK cell cytotoxicity, showing decreased expression of granzyme B and IFN-γ in rapamycin-treated mice compared to controls (30, 39–42).

In this respect, many investigations have been developed to explore the activation-specific metabolic changes of NK cells. Almost all evaluate the relationship between cell metabolism and antitumor response, demonstrating that tumor NK cells present metabolic dysfunction (reduced glycolytic capacity and OXPHOS pathway) and decreased antitumor response (43). Most of these studies are consistent with the metabolic changes observed in efficient tumor killing, highlighting the important role of the mTOR pathway and increasing glycolysis for NK cell cytotoxicity (44).

The NK cell phenotype is influenced by its microenvironment; in this way, different factors, such as nutrient availability, can modulate their effector functions (36). An example is the excess nutrients (fatty acids and glucose) observed in different metabolic diseases. *In vitro* studies have shown that fatty acid excess increases lipid internalization of human blood NK cells and is directly related to decreased antitumor capacity (44).

Nutrient deprivation also influences the functional capacity of these cells. Different factors in the tumor microenvironment are related to these metabolic changes. Tumor cells require high amounts of energy for accelerated proliferation. These demands increase the cells’ metabolic capacity and decrease nutrient availability in the tumor microenvironment. As a result, tumor-infiltrating NK cells may be glucose and oxygen-deprived, leading to their inability to turn on mTORC1 for effector functions (45–47).

In response to energetic stress, NK cells activate other metabolic regulators to adapt to changing nutrient and inflammatory settings during an immune response (17). One of the stress-activated metabolic regulators is hypoxia-inducible Factor-1α (HIF1α), an evolutionarily conserved transcription factor. Which, in an oxygen-dependent manner, stabilizes and promotes the expression of genes directly related to cellular metabolism (30, 48).

An *in vitro* study in human NK cells demonstrated that HIF1α inhibition enhanced degranulation and IFN-γ and TNF-α production. Similarly, *HIF1A* expression in human tumor-infiltrating NK cells is negatively associated with their antitumor function, highlighting the potential function of HIF1α inhibitors in cancer therapy (49). PGC1α (peroxisome proliferator-activated receptor gamma coactivator 1-alpha) is another metabolic regulator that functions as a transcriptional coactivator of mitochondrial function-related genes and is required for cytotoxicity and cytokine production in NK cells (17).

## Immunometabolism of NK cells in viral infections

Although the antitumor response of NK cells is one of their most important functions, their antiviral response is also essential but less studied regarding immunometabolism. Over the years, studies have confirmed the association between immunity and metabolism, showing that metabolic pathways can control the innate and adaptive host responses to infection (24, 50–53). During viral infection, NK cells are exposed to various stimuli including inflammatory cytokines and viral ligands; however, the changes these signals induce in the metabolic profile of NK cells are poorly characterized.

Viruses are intracellular parasites that depending on host metabolism to maintain viral replication. This generates important metabolic changes in infected cells to provide the specific substrates required at high levels during virion production (54). In the last decade infections caused by viruses such as human cytomegalovirus (HCMV), adenovirus, hepatitis C virus (HCV), Herpes Simplex Virus (HSV), and Epstein-Barr virus (EBV), have been investigated regarding immunometabolism, demonstrating an increase in glucose uptake, glycolytic enzyme expression, lactic acid production, and fatty acid synthesis, in infected cells for viral replication (54–61). Some viruses, such as HIV-1, preferentially infect activated cells that are metabolically active (62), emphasizing the importance of understanding cell metabolism as a potential target in treating viral infections.

The most common model for studying NK cells in a viral infection context is cytomegalovirus (CMV). This model is characterized by the proliferation and function of virus-specific NK cells that eliminate infected cells (63). In response to HCMV, NK cells activate the NKG2C receptor and rapidly expand and persist; these cells display enhanced responses (64). In mice, during the first two days of CMV infection, NK cells are activated through a strong host-cytokine response. After day 2, NK cells recognize the viral-encoded protein m157 on infected cells through the Ly49H activation receptor, triggering cytotoxicity and cytokine production that can modulate subsequent immune responses (65).

*In vivo* studies in a mouse model of CMV infection demonstrated enhanced aerobic glycolysis in NK cells by upregulation of many genes required for this metabolic pathway, increased oxygen consumption rate (OCR), and extracellular acidification rate (ECAR). Also, NK infected with CMV showed higher expression of nutrient transporters such as CD98, GLUT1, and CD71, which support nutrient uptake for ATP production from metabolic pathways (63, 66, 67).

The importance of metabolism in NK cell functions has also been studied using metabolic inhibitors or genetic deletions in murine models. CMV-infected mice show that glycolysis inhibition by 2-DG impaired NK-mediated target clearance and significantly increased the susceptibility of mice to infection (68). Similarly, mice knockout for lactate dehydrogenase A (LDHA), a central enzyme in glycolysis signaling, rapidly succumbed to CMV infection compared to controls, demonstrating that induction of aerobic glycolysis in NK cells is vital for an effective antiviral host immune response (63).

Research of other viral infections is less extensive but suggests metabolic changes similar to those reported during CMV infection. A study conducted on patients with EBV infection demonstrated upregulation of the PI3K/Akt/mTOR pathway, which has been reported in other viral infections and could indicate a general response to viral infection (56).

Most recently, Littwitz-Salomon et al. evaluated the metabolic changes of NK cells against retrovirus infection (Friend virus) in a murine model, showing that NK cells from infected mice significantly increase in size. This finding correlates with increased cytotoxicity and expression of activation markers such as CD69 and nutrient transporters, specifically amino acids and iron (CD98 and CD71, respectively) (66).

Although both metabolic pathways, glycolysis and OXPHOS, are important for NK cell function. During activation, NK cells prefer glycolytic metabolism because it is a faster way to provide energy and compensate alterations in other metabolic pathways such as OXPHOS. This phenomenon was described as “metabolic flexibility” by Mah-Som et al. (2021) in a murine model of CMV infection, where it was observed that defects in OXPHOS due to cyclooxygenase-2 (COX2) deletion promote the upregulation of glycolytic enzyme genes: *LDHA*, glyceraldehyde-3-phosphate dehydrogenase *(GAPDH)*, pyruvate kinase muscle isozyme *(PKM)*, enolase 1 *(ENO1)*, phosphoglycerate kinase 1 *(PGK1)*, aldolase A *(ALDOA)* (69). These findings show that NK cells respond quickly to metabolic changes to maintain proper function.

Different factors directly influence the function of NK cells in viral infections, like in the tumor microenvironment. During an acute viral infection, the virus-infected cells substantially increase their metabolic capacity to improve the energy requirements for viral replication. As a result, this inflammation site can become nutrient depleted, limiting the glucose levels available for immune cells in the local microenvironment (30). This scenario of nutrient depletion has been observed in human viral infections such as SARS-CoV2, where a decrease in serum iron levels in patients is associated with an increased risk of severe COVID-19, acute organ failure, and a worse prognosis (70–74).

Therefore, these nutrient-deficient environments may play an important role in unbalancing NK cell functions. In this context, cells activate different metabolic regulators, such as HIF1α, to adapt to new conditions. Murine models demonstrate that CMV infection upregulates HIF1α to protect against morbidity, control viral load, and support the survival of NK cells by providing optimal glucose metabolism during pathogen infection (64).

Some viral infections persist for days or even weeks, maintaining constant antigenic stimulation by the presence of viral ligands or inflammatory cytokines at the site of infection. This constant stimulation may be the cause of decreased NK cell function. In this regard, Felices et al. demonstrated that continuous chronic stimulation of NK cells with IL-15 (9 days) resulted in functional changes in NK cells, consistent with exhaustion, displaying markedly diminished cytolytic and inflammatory function (75).

**Figure 1:**
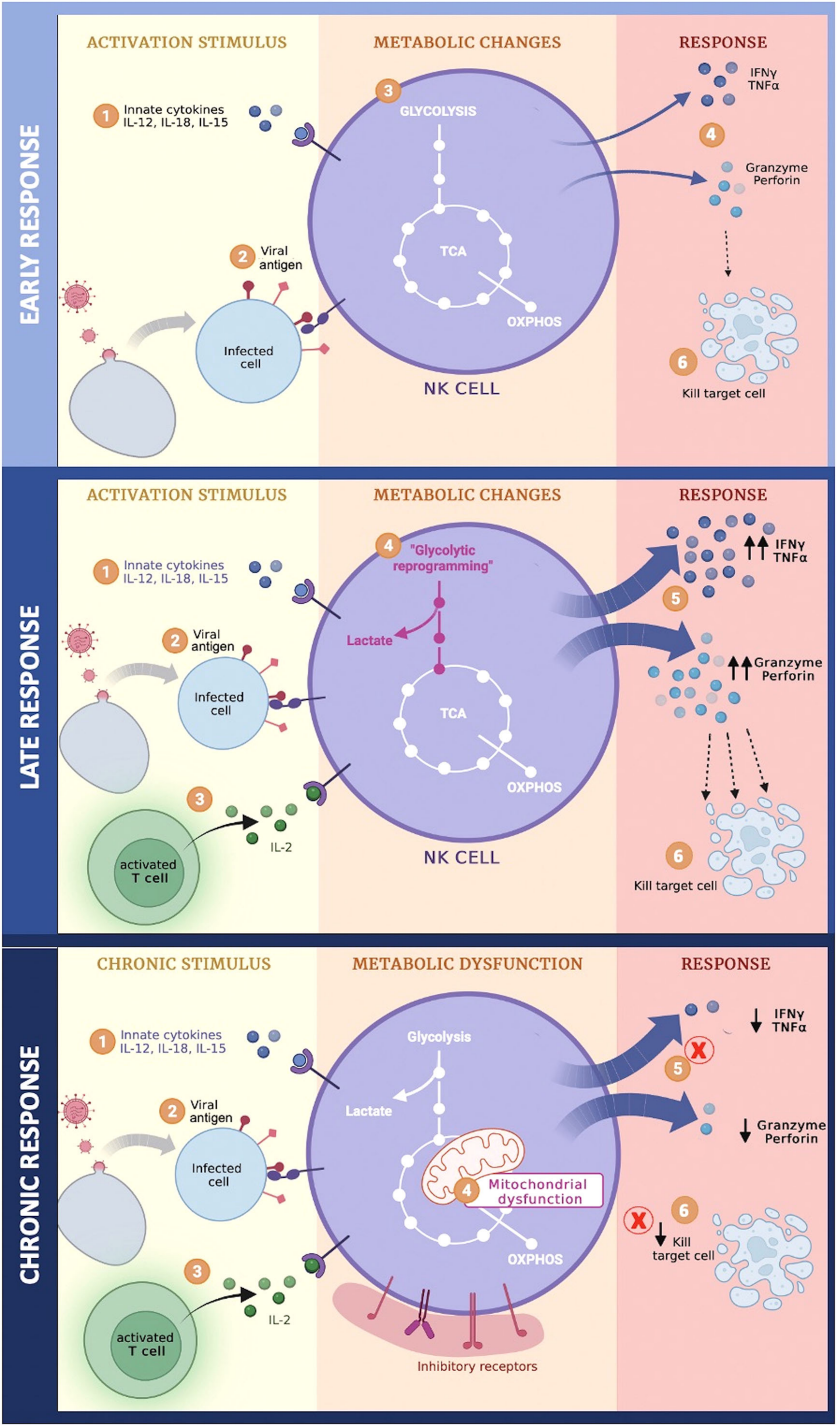
Metabolic changes of NK cells during different phases of viral infection.

In summary, during the early phase of acute viral infection, NK cell activation is mediated by binding of ligands to activating receptors and cytokines receptors. This stimulation does not produce significant metabolic changes, the basal metabolic profile can provide the energy required to support their effector functions against the virus; however, the late phase of the acute infection generates important changes such as increased glycolysis and OXPHOS trying to maintain the energy supply for the continuous stimulus from both the viral antigen, as well as cytokines secreted by innate and activated T cells, thus increasing its cytotoxic capacity. Subsequently, if stimulation continues, NK cells acquire an exhausted phenotype characterized by metabolic dysfunction, decreased cytotoxicity, and increased expression of inhibitory receptors, such as Tim-3, PD-1, and NKG2A, on their surface (**Figure 1**) (27, 74).

The prolonged stimulation of NK cells has been evaluated in metabolic diseases such as diabetes mellitus, obesity, or hyperlipidemia, where there is an excess in the availability of nutrients. The excess of glucose and lipids participate in the constant activation of NK cells causing their exhaustion and dysfunctional capacity (76–78). In this scenario, people with metabolic diseases have high-risk to develop complications during viral infections (79–81).

## Metabolic changes of NK cells during SARS-CoV2 infection

The current Coronavirus Disease 2019 (COVID-19) pandemic caused by SARS-CoV-2 generated emerging information based on the dysregulation of the innate and cell-mediated immune response observed during this viral infection (82).

Some studies reported that patients with COVID-19 present a significant decrease in total lymphocyte count in peripheral blood, mainly in cytotoxic lymphocytes (CD8 T and NK cell). These cell populations present functional alterations directly related to disease progression. In patients with “moderate” disease, an increase in cytotoxic mediators such as granzyme A and perforin has been reported in cytotoxic lymphocytes (NK and CD8 T cells). Several authors suggest this increase in NK cell cytotoxicity is due to a compensatory response for the lymphocyte depletion (83–88).

In recovered patients the total lymphocyte counts increase and the production of cytotoxic mediators (granzyme A and perforin) by NK cells decreases, thus recovering homeostasis. However, in patients with severe disease this lymphocyte recovery is absent, and the production of cytotoxic mediators continues to be elevated, directly contributing to tissue damage and a worse prognosis. In addition, it has been observed that CD8+ and NK cells of severe patients present increased expression of inhibitory markers, such as PD-1, LAG3, TIGIT and NKG2A, characteristic of an “exhausted” phenotype, unable them to resolve the infection (4, 82, 89–91).

Given that the functional capacity of these cells is directly related to their cellular metabolism, it is interesting to evaluate the participation of cellular metabolism in the functional decline reported in these cells.

Transcriptome study in mononuclear cells from COVID-19 patients revealed the upregulation of several metabolic genes associated with the mitochondrial respiratory electron transport chain (ETC), glycolysis and gluconeogenesis (92).

The main cell populations affected by SARS-CoV-2 infection are cytotoxic lymphocytes (NK and CD8 T cells). Metabolic changes in CD8+ T cells are currently identified in COVID-19 patients showing a decreased ability to upregulate cellular metabolism by decreased activation of mTOR signaling early after activation, coupled with additional defects in metabolic reprogramming to glycolysis (93).

The low numbers and functional capacity of NK cells in peripheral blood are related to the migration of these cells into the lung tissue, where they actively participate in the resolution of viral infection. Few investigations have been performed to evaluate metabolic changes in NK cells during SARS-CoV-2 infection. Even though, an important study evaluated the NK cells from lung tissues of 19 deceased COVID-19 patients. In this study, high levels of some metabolic pathways (glycolysis, inositol phosphate and glycerolipid metabolism) and two enzymes in glycerolipid metabolism pathway (lipin 1 and lipin 2) were found. These metabolic changes may contribute to enhance NK cell functions, such as chemotaxis, degranulation and cytotoxicity (94).

Large parts of the population at risk for COVID-19 have metabolic diseases such as diabetes, overweight/obesity, and hyperlipidemia, among others. These pathologies share a common scenario: an alteration in the availability of nutrients (fatty acids or glucose) which have been shown to significantly impact immune system cell function, altering cell metabolism and directly affecting their effector function (44). Therefore, people with metabolic conditions that compromise overall metabolic health have a greater risk of developing a severe infection (95).

Several metabolic changes have been identified during SARS-CoV-2 infection. Patient metabolism during the disease is quite dynamic, showing deep alteration of lipoprotein particles (increased triglyceride content and VLDL) and a hypermetabolic state, which may be a major contributing factor to the extraordinary ventilatory, and oxygenation demands in patients with COVID-19. This immune-metabolic crosstalk during COVID-19 progression suggests potential use of metabolites to control the disease through direct modulation of specific steps of lipid metabolism, opening avenues for the development of metabolic-based therapies (96, 97).

## Improvement of NK cell function by metabolism modulation

The multiple functional uses of NK cells have been explored in different ways to activate them for clinical applications. These efforts have concentrated on blocking NK cell inhibitory receptors with antibodies, transferring allogeneic-activated NK cells, or engineering these cells (98).

More recently, the relationship between metabolism and cytotoxic function of NK cells is clear and represents an interesting approach to developing strategies that address metabolic reprogramming of NK cells in the context of immunotherapy of different diseases where NK cell function is altered.

It has been known that NK cell functions are defective in diverse viral infections and cancers, conditioning their progression (10). The great majority of studies on metabolic manipulations have been performed *in vitro* and murine models with different drugs (59).

A recent approach establishes the possibility of using 2-DG, a glycolysis inhibitor, as an antiviral treatment. This finding is supported by the knowledge that infected cells increase the glycolytic pathway during viral replication. An *in vivo* study evaluated the consequences of 2-DG therapy in the inflammatory phase of HSV infection, demonstrating a decrease in inflammatory lesions. However, limit glucose source in mice during the acute phase of the infection may cause susceptibility to lethal outcome (99).

In India, some clinical trials with oral or intravenous 2-DG are being evaluated in humans as a treatment for SARS-CoV-2 infection. The therapy was shown to be safe and well tolerated. Some benefits demonstrated are normalization of vital signs and clinical recovery (35, 100).

Another potential metabolic target is the inhibition of fatty acid and glutamine metabolism, which are essential for viral replication. In murine models, the *in vivo* inhibition of these metabolic pathways, results in diminished inflammation caused by viral infection (flavivirus and Sindbis virus, respectively) (101, 102). Although the human trials and murine models have shown to be a potential treatment of viral infections, more research is needed to rigorously evaluate the consequences of these metabolic inhibitors.

Some others efforts in immunotherapy have focused on engineering NK cells to maintain mTOR complex activity, mediate NK cell activation, and improve their antiviral response (45, 103). Another metabolism regulator is cMyc, which regulates the expression of the metabolic machinery required to support elevated rates of glycolysis and OXPHOS. Strategies that stabilize mTOR and cMyc in NK cells might provide a metabolic advantage for these cells by sustaining glucose metabolism and enhancing their cytotoxic antiviral activity (45, 104).

Another approach is microenvironment modifications to change cell metabolism. An example is lactate accumulation, which has been identified as a negative regulator of NK cell function. Some compounds, such as metformin and dichloroacetate (DCA), have been identified as candidate drugs for enhancing NK cell cytotoxicity and the effects of antiviral immunotherapy. Metformin directly activates mTOR signaling and DCA improving oxidative metabolism by PDH Kinases (PDKs) inhibition (98, 105).

More recently, the study of microRNAs (miRNAs) as cellular metabolism modifiers has gained interest in immunometabolism. miRNAs are a class of non-coding RNAs that regulate endogenous gene expression at the post-transcriptional level. It has been demonstrated that these can govern changes in interconnected metabolic pathways (106, 107). Most of the information available on miRNAs and metabolism has been evaluated in T cells, providing insights into other cell populations, such as NK cells.

Energy requirements differ during cell differentiation according to the needs and functions of the specific cell subset. Different cellular process (activation, proliferation, and apoptosis) in T lymphocytes and NK cell are fundamentally regulated by the expression of a core set of miRNAs, termed “immuno-miRs”. Highlighting the relevance of miRNAs as modulators of metabolic and cellular processes (108, 109).

Some of the miRNAs which regulate cellular metabolism have been identified in T cells, essential for the activation and function of these cells. The most common routes targeted by miRNAs to modulate T cell metabolism includes interfering with glycolysis and key metabolic regulators such as mTOR, AMPK, Myc, and HIF-1α. For example, miR-214, miR-19b, and miR-21 act as inhibitors of PTEN, a potent inhibitor of the PI3K/AKT/mTOR pathway (107). Identical mechanisms are predicted to be operative in NK cells; therefore, miRNAs are proposed as a therapeutic tool in diseases that involve dysfunctional cell metabolism. However, future studies are needed to validate this claim (110).

**Figure 2:**
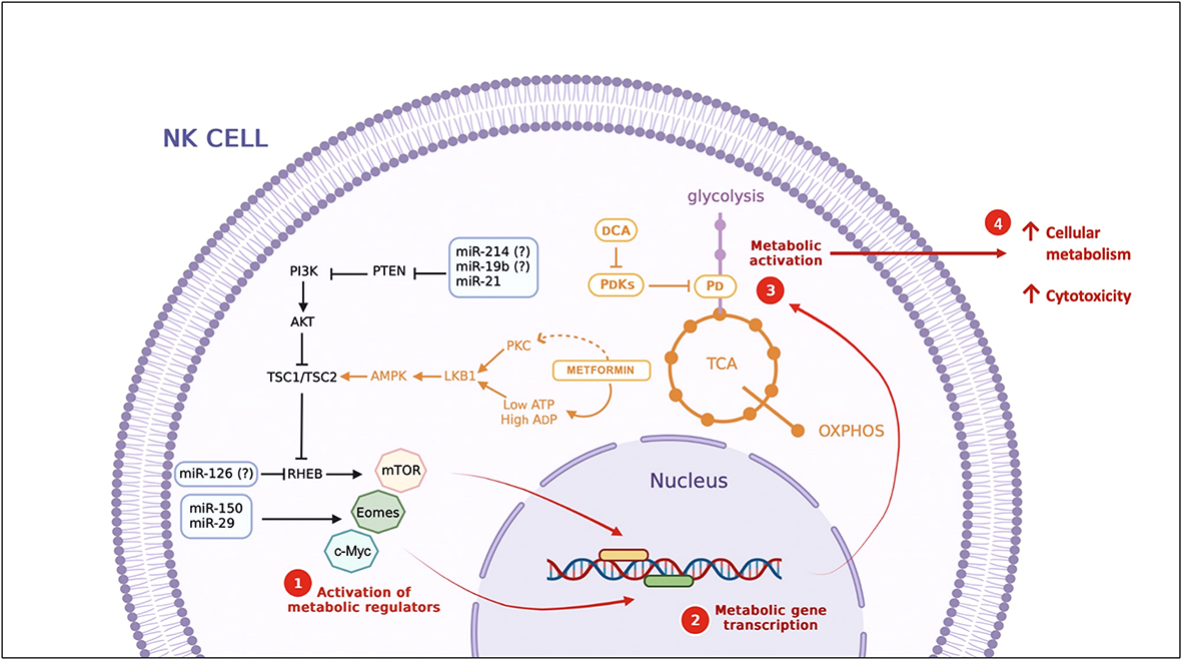
Strategies to modulate cellular metabolism. miRNAs and compounds with metabolic modulator activity. miRNAs identified in T cells, DC and NK cells, some of them (miR-21, miR-150 and miR-29) (113) also have been identified during NK cell activation. *(?) miRNAs identified in T cells, DCA, dichloroacetate; PDKs, PDH Kinases; PDH, Pyruvate dehydrogenase; TCA, Tricarboxylic Acid Cycle.*.

On the other hand, miRNAs that directly regulate the PI3K/AKT/mTOR signaling pathway have also been identified in innate immunity cells. miR-126 regulates dendritic cell survival and function by inhibiting TSC1 (mTOR inhibitor). Also, miR-150 targets the transcription factors Eomes and c-Myc promoting NK cell maturation, a process characterized by substantial energetic changes (110–112) (**Figure 2**).

In cancer, glucose metabolism emerges as one of the critical points for miRNA actions by promoting or inhibiting aerobic glycolysis. Tumor-suppressive miRNAs that inhibit metabolic pathway enzymes are currently used in clinical trials to treat cancer (114). In a viral infection context, these same principles could be applied to reprogram NK cell metabolism to restrict viral replication and improve clinical outcomes. Glycolysis is an essential metabolic pathway for virion production; therefore, blocking this metabolic pathway in infected cells could directly affect viral replication. On the other hand, the metabolic defects present in NK cells could be regulated by miRNAs to improve their cytotoxic function against infected cells. Several miRNAs have been reported to repress key steps in the glycolytic pathway, which could be identified as potential therapeutic agents (**Figure 2**) (115). Therefore, metabolism modulation is proposed as a therapeutic to improve NK cell cytotoxicity in viral infections (116). Indeed, more studies are needed to explore the metabolic changes in NK cells in different clinical stages of SARS-CoV2 infection.

## Conclusions and perspectives

NK cells are the first line of defense and play an important role in the resolution of viral infections. Currently, cellular metabolism has been identified as responsible for the functional capacity of these cells, and it has been observed that alterations in the metabolic profile of NK cells directly influence the proliferation, activation, and cytotoxic capacity of these cells.

Metabolic changes in NK cells, which could be associated with viral control and clinical outcomes, have been reported in viral infections. Recently, the “new” viral infections and their clinical presentations have become a challenging to search for novel therapeutic strategies to improve public health. In the current pandemic of COVID-19, important functional alterations in NK cells directly related to the severity of the disease have been reported. These functional alterations could be due to metabolic alterations in these cells. In this sense, NK cell metabolism manipulation could be used to improve their cytotoxic and cytokine production functions.

To date, different protocols have been developed trying to manipulate the metabolism of NK cells and thus improve their functional capacity and antiviral response. Although it is a promising future strategy for treating viral infections, it remains an underexplored area of immunometabolism, requiring further scientific investigations.

## Author contributions

KO-E was responsible for designing the content structure, collecting literature, and writing the original draft. AR-T supervised the whole manuscript, made great contributions in redaction, editing and design the work. All authors contributed to the article and approved the submitted version
